# Specific recognition of guanines in non-duplex regions of nucleic acids with potassium tungstate and hydrogen peroxide

**DOI:** 10.1093/nar/gku1025

**Published:** 2014-10-29

**Authors:** Wuxiang Mao, Xiaowei Xu, Huan He, Rong Huang, Xi Chen, Heng Xiao, Zhenduo Yu, Yi Liu, Xiang Zhou

**Affiliations:** College of Chemistry and Molecular Sciences, Key Laboratory of Biomedical Polymers of Ministry of Education, State Key Laboratory of Virology, Wuhan University, Hubei, Wuhan 430072, P. R. China

## Abstract

Structural features of nucleic acids have become an integral part of current biomedical research. Highly selective and readily performed methods with little toxicity that target guanosines in non-duplex nucleic acids are needed, which led us to search for an effective agent for guanosine sequencing. Treatment of DNA or RNA with potassium tungstate and hydrogen peroxide produced damaged guanosines in DNA or RNA sequences. The damaged guanosines in non-duplex DNA could be cleaved by hot piperidine. Similarly, damaged guanosines in non-duplex RNA could be cleaved by aniline acetate. We could identify structural features of nucleic acid using this strategy instead of dimethyl sulphate and Ribonuclease T1.

## INTRODUCTION

Nucleic acids play a critical role in many physiological processes *in vivo* and can be mutated and damaged by various physical, chemical and biological factors (1,2). The mutation and damage of nucleic acids often leads to errors (3). Various chemical and biological methods have been explored to investigate nucleic acid degradation (4–6). Natural protein nucleases catalyze DNA/RNA degradation (7,8), and certain types of chemical nucleases also have the ability to cleave nucleic acids via hydrolysis and oxidation (9–10,13). For example, the guanines in a double helix-coil junction in DNA can be cleaved by a trinuclear copper complex and hot piperidine (11–12); guanosines in non-duplex DNA regions can be recognized by manganese corroles via active oxygen transfer (9); and the guanine structure of nucleic acids can be recognized by nickel complexes (13). Nucleic acids are known to include non-canonical motifs, such as hairpins, mismatches, G-quadruplexes, terminal loops and bulges. Non-duplex DNA structures are considered to be more prone to mutation during DNA replication than duplex DNA, which may play a critical role in the chromosomal alterations found in cancer cells (14). Therefore, it is necessary to develop a method to target these non-duplex structures and to specifically cleave these portions out of the DNA.

Due to its electron-rich properties, guanine is the most readily oxidized nucleobase of the four natural bases (15). The chemical modification of guanine has been linked to aging, cancer and degenerative diseases (16–18). A wide range of aqueous oxidation systems, including Mn(II), Ni(II) and Cu(II) compounds and certain metal ligands such as porphyrins, corroles and other planar compounds, have been known to react with the electron-rich guanine (9–13). However, these metal complexes are not easily obtained because of their complicated synthesis and troublesome screening. In addition, Ribonuclease T1 (RNase T1) from *Aspergillus oryzae* is an endoribonuclease that can hydrolyze residues following guanine (19). However, RNase T1 is very expensive and is too large to approach the guanine residues that would normally be recognized as substrates. The classical alkylating agent dimethyl sulphate (DMS) can be rapidly applied to alkylate guanine residues, and the reaction can be halted with ethanethiol (20). This classical Maxam–Gilbert chemical method for DNA sequencing has provided us with useful information on guanine residues in DNA sequences. However, DMS is highly toxic, which could lead to chromosomal alterations, and ethanethiol is also poisonous and effluvial. Herein, we have found a less toxic chemical agent—potassium tungstate (21) and hydrogen peroxide—to selectively cleave guanosine units in single-stranded nucleic acids, including non-duplex DNA structures and non-duplex RNA structures.

## MATERIALS AND METHODS

### Materials

All reagents were used as supplied from Sigma-Aldrich Corporation. The HEX-labeled DNA/RNA was purchased from Takara Biotechnology (Dalian, China). Mass Spectrometer (MS) was recorded on a Brucker Daltonics APE XII 47e via using an electrospray ionization (ESI)-positive mode.

### Preparation of DNA containing several structures

DNA sequences containing several structures were annealed at high concentration in 10 mM Tris–ethylenediaminetetraacetic acid (EDTA) (pH 7.4) and 100 mM NaCl by heating to 95°C followed by slow cooling.

G-quadruplex DNA was prepared in 10 mM Tris–EDTA (pH 7.4), 200 mM KCl and 1 M NaCl by heating DNA to 95°C and then slowly cooling to room temperature.

### Cleavage of DNA/RNA

To 6-μl Na_2_HPO_4_-NaH_2_PO_4_ buffer solution (pH 7.0, 10 mM), 2 μl DNA (10 μM), 1 μl K_2_WO_4_ (50 mM) and 1 μl H_2_O_2_ (500 mM) were added, respectively. The mixture was incubated at 37°C. After 2 h, ethanol (1 ml, 100%) and 10 μl CH_3_COONa-CH_3_COOH buffer (1 M, pH 5.0) were added to this mixture. This mixture was frozen at −80°C for 1 h and then centrifuged for 20 min. After removal of upper solution, the DNA precipitate was vacuum dried, then redissolved in 10% piperidine and heated at 90°C for 30 min. After the piperidine treatment, the DNA was precipitated again as the step mentioned above. After dried by vacuum, the DNA precipitate was dissolved in 80% formamide for further examination on acrylamide gels.

To 6-μl Na_2_HPO_4_-NaH_2_PO_4_ buffer solution (pH 7.0, 10 mM), 2 μl RNA (10 μM), 1 μl K_2_WO_4_ (50 mM) and 1 μl H_2_O_2_ (500 mM) were added, respectively. The solution was incubated at 37°C. After 2 h, 1 ml ethanol ethanol (100%) and 10 μl CH_3_COONa-CH_3_COOH buffer (1 M, pH 5.0) were added to this mixture. The mixture was then frozen at −80°C for 1 h and centrifuged for 20 min. After removal of upper solution, the RNA precipitate was dried by vacuum, then redissolved in 20 μl aniline acetate (1 M, pH = 4.5) and heated at 60°C for 20 min in the dark. After the aniline treatment, the RNA was precipitated as mentioned above. After dried by vacuum, the RNA precipitate was dissolved in 80% formamide for further examination on acrylamide gels.

### Cleavage of denatured DNA (22)

To 24 μl of formamide, DNA (2 μl, 10 μM), 2 μl K_2_WO_4_ (300 mM) and 2 μl H_2_O_2_ (3 M) were added, respectively. The solution was incubated at room temperature. After 5 min, 1 ml ethanol (100%) and 10 μl CH_3_COONa-CH_3_COOH buffer (1 M, pH 5.0) were added. The denatured DNA was precipitated as mentioned above.

### Preparation of G-ladder (23)

In total, 2 μl DNA (10 μM), 10 μl Tris–EDTA buffer (100 mM, pH 7.8) and 88 μl distilled water were mixed to the G reaction tube, while 40 μl distilled water, 20 μl CH_3_COONa-CH_3_COOH buffer (1 M, pH 5.0) and 40 μl ethanethiol were combined in another tube to prepare DMS stop buffer. Then, 2 μl DMS was added to the G reaction tube. The mixture was incubated at room temperature for 5 min. The DMS stop buffer and the prechilled 100% ethanol were then added to the G reaction tube and mixed thoroughly. The mixture was frozen at −80°C for 1 h and centrifuged for 20 min. After removal of upper solution, the DNA precipitate was dried by vacuum, redissolved in 10% piperidine and heated at 90°C for 30 min. After the piperidine treatment, the DNA was precipitated as described above. After dried by vacuum, the DNA precipitate was dissolved in 80% formamide for further examination on acrylamide gels.

### Photooxidation of DNA by methylene blue (24,25)

A 5 μl solution containing 1.5 M NaCl, 0.5 M Tris–HCl buffer (pH 8.5), 2 μl DNA (10 μM) was added to 20 μl 0.1% methylene blue in water. This reaction was irradiated by a high pressure mercury lamp (GHg-50A) at room temperature for 15 min. O_2_ was bubbled through the mixture during the course of the reaction. After 15 min, the DNA was precipitated as described above.

### RNA digestion by RNase T1 (26)

RNA was incubated at 4°C with RNase T1 (0.01 unit) in the presence of 200 mM NaCl, 5 mM MgCl_2_ and 10 mM Tris–HCI (pH 7.6). After 10 min, 1 ml ethanol ethanol (100%) and 10 μl CH_3_COONa-CH_3_COOH buffer (1 M, pH 5.0) were added to this mixture. The mixture was then frozen at −80°C for 1 h and centrifuged for 20 min. After removal of upper solution, the RNA precipitate was vacuum dried. Finally, the RNA precipitate was dissolved in 80% formamide for further examination on acrylamide gels.

### RNA annealing (26)

An RNA solution containing 10 mM MgCl_2_, 100 mM NaCl and 10 mM potassium phosphate (pH 7.0) was heated to 50°C for 5 min and allowed to cool slowly to ambient temperature (1.5 h) before further use.

### Nucleoside studies

A 50 ml solution of dG (1 mmol, 285 mg) in distilled water was incubated with K_2_WO_4_ (1 mmol, 326 mg) and H_2_O_2_ (1 ml) at room temperature. After the dG was completely consumed by oxidant according to TLC monitoring, Na_2_EDTA (744 mg) was added to quench further oxidation. Finally, the reaction products were analyzed by mass spectrometry.

## RESULTS

### Oxidation of single-stranded DNA by K_2_WO_4_ or H_2_O_2_

**Scheme 1.**
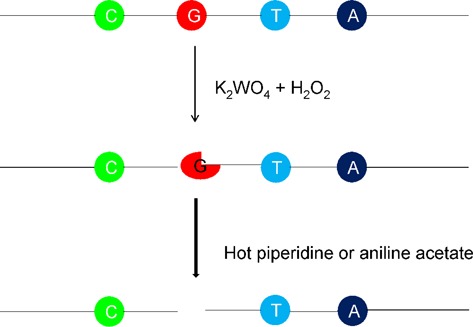
Schematic illustration of nucleic acid sequences oxidized by oxidant and then cleaved by hot piperidine or aniline acetate.

The cleavage procedure is illustrated in Scheme 1, where K_2_WO_4_ reacts with hydrogen peroxide to form K_2_W(O_2_)_4_ (27,28), which can specifically oxidize guanosine; the DNA sequence, including the oxidized guanosine, can then be cleaved by hot piperidine at the guanosine residue. Similarly, an RNA sequence that includes oxidized guanosine can be cleaved by aniline acetate at pH = 4.5 (26). Molecular modeling calculations have demonstrated that oxidants can more readily approach the guanine residues in non-duplex DNA structures.

**Figure 1.**
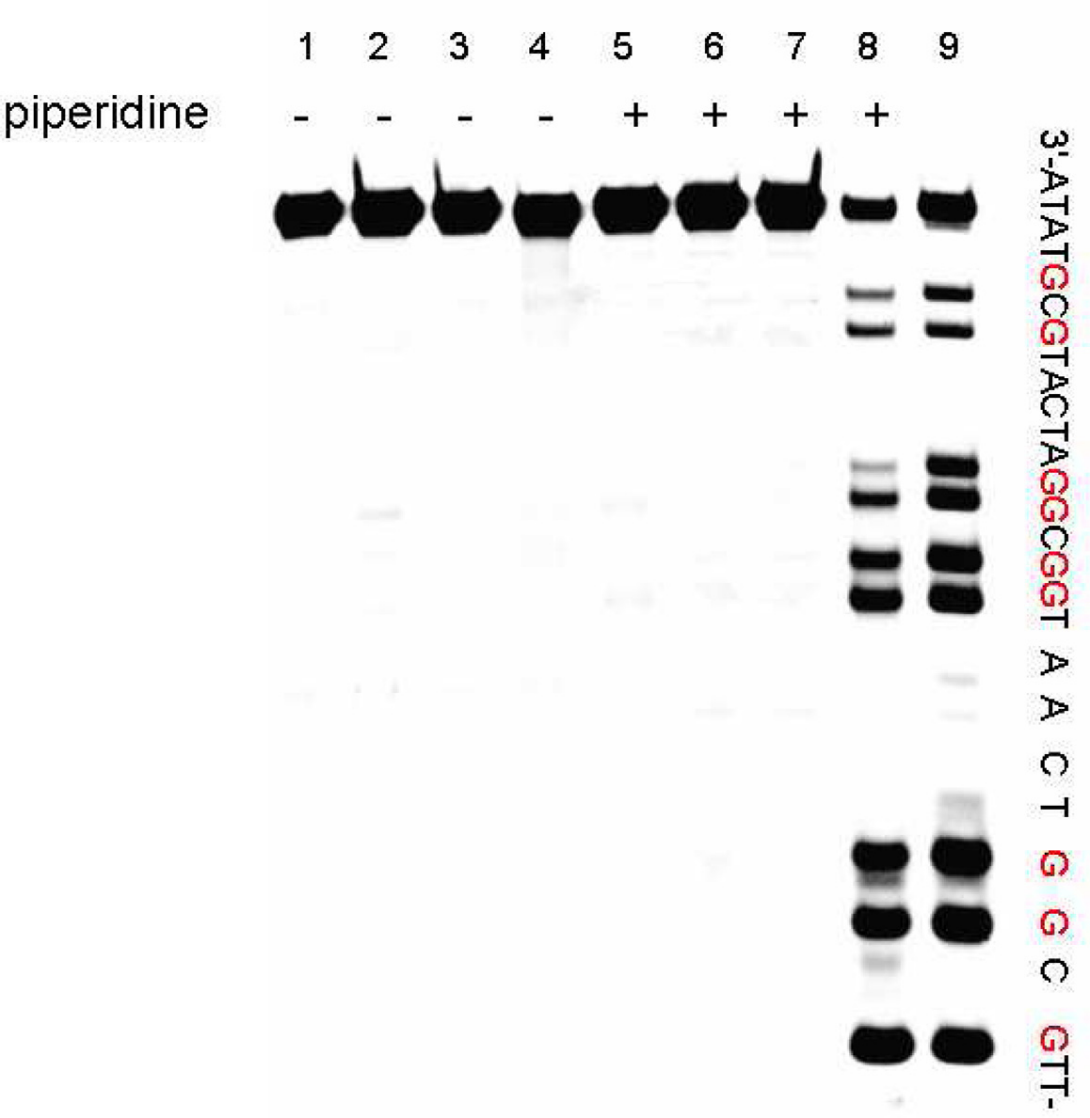
Polyacrylamide gel electrophoresis experiments showing cleavage products of 5’-HEX-labeled ODN1 (20 pmol) incubated with the K_2_WO_4_ and H_2_O_2_ for 2 h in sodium phosphate (10 mM, pH 7.0) at 37°C. Lane 1, 5: ODN 1 alone; lane 2, 6: ODN 1 with 5 mM K_2_WO_4_; lane 3, 7: ODN 1 with 50 mM H_2_O_2_; lane 4, 8: ODN 1 with 5 mM K_2_WO_4_ and 50 mM H_2_O_2_; lane 9: G-ladder (DMS treated); lane 5–8: After 10% piperidine treatment at 90°C for 30 min.

**Figure 2.**
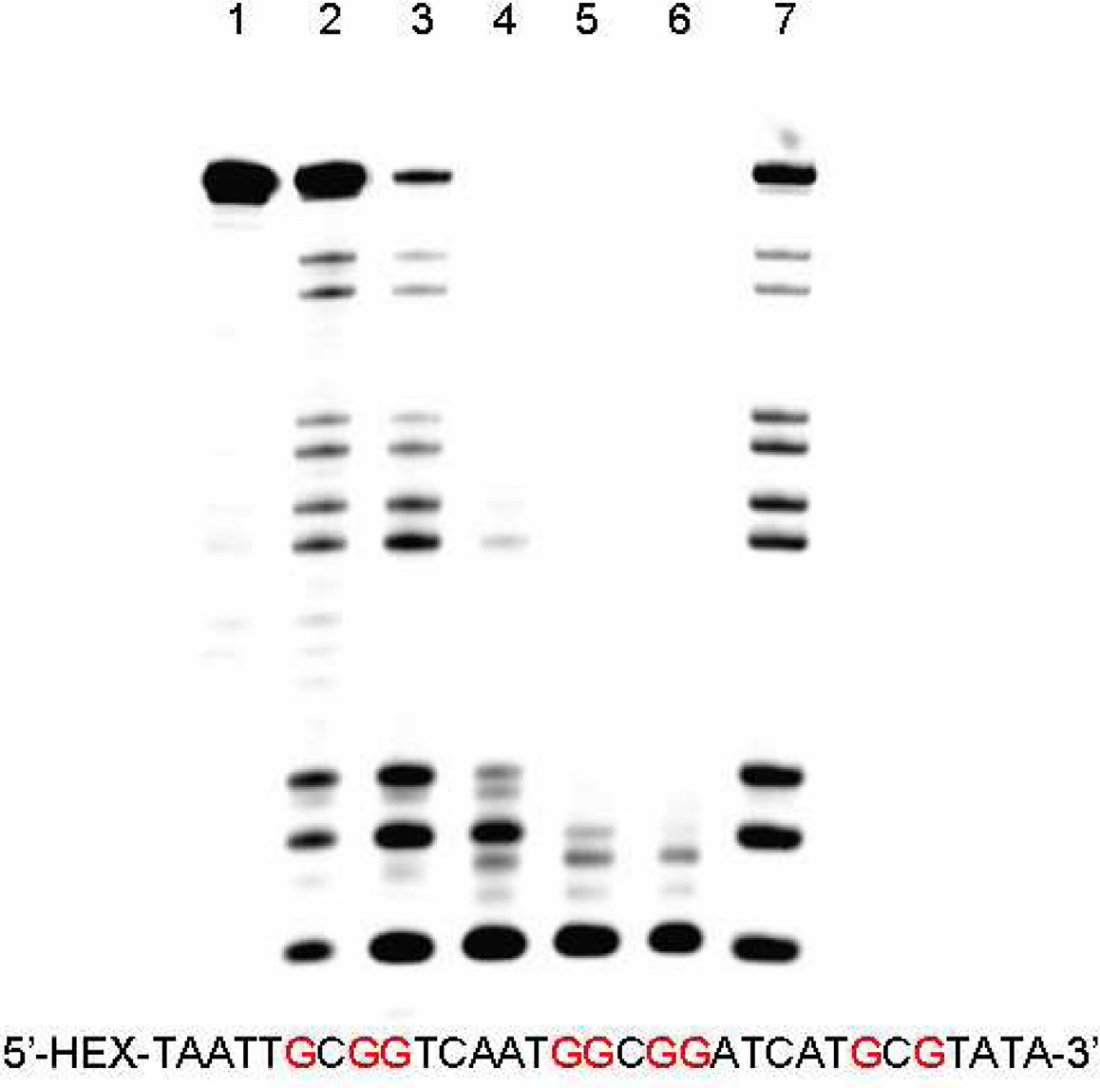
Polyacrylamide gel electrophoresis analysis of ODN 1. Treat the ODN 1 (20 pmol) with piperidine at 90°C for 30 min, after oxidized by different concentration of K_2_WO_4_ and H_2_O_2_. The reacting concentration of K_2_WO_4_ in lanes 1–6 was 0, 5, 10, 15, 20 and 25 mM, respectively. The reacting concentration of H_2_O_2_ in lane 1–6 was 0, 50, 100, 150, 200 and 250 mM, respectively. Lane 7: G-ladder (DMS treated).

**Figure 3.**
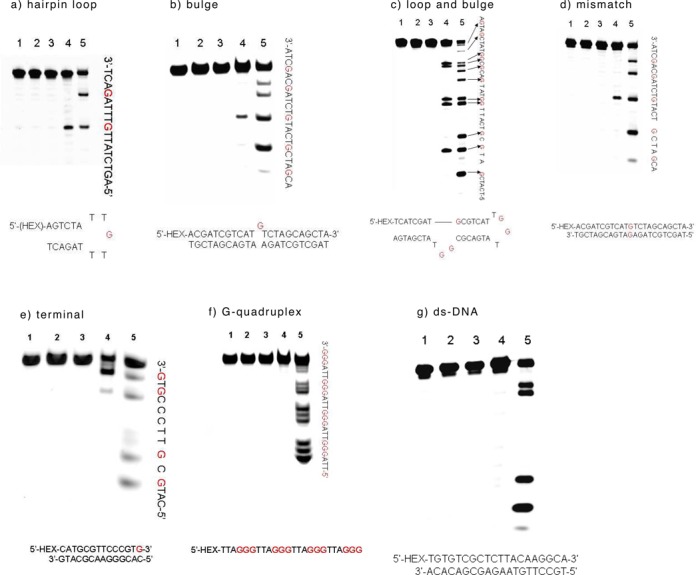
Polyacrylamide gel electrophoresis analysis of DNA. Treatment of the DNA (20 pmol) with piperidine at 90°C for 30 min after oxidized by 5 mM K_2_WO_4_ and 50 mM H_2_O_2_ for 2 h. Lane 1: DNA alone; lane 2: DNA with 5 mM K_2_WO_4_; lane 3: DNA with 50 mM H_2_O_2_; lane 4: DNA with 5 mM K_2_WO_4_ and 50 mM H_2_O_2_; lane 5: G-ladder (DMS treated). (**a**) Cleavage of the hairpin loop structure by K_2_WO_4_ and H_2_O_2_. (**b**) Cleavage of the bulge structure by K_2_WO_4_ and H_2_O_2_. (**c**) Cleavage of the hairpin loop and bulge structure by K_2_WO_4_ and H_2_O_2_. (**d**) Cleavage of the mismatch structure by K_2_WO_4_ and H_2_O_2_. (**e**) Cleavage of the terminal structure by K_2_WO_4_ and H_2_O_2_. (**f**) Cleavage of the G-quadruplex structure by K_2_WO_4_ and H_2_O_2_. (**g**) Cleavage of the ds-DNA structure by K_2_WO_4_ and H_2_O_2_.

**Figure 4.**
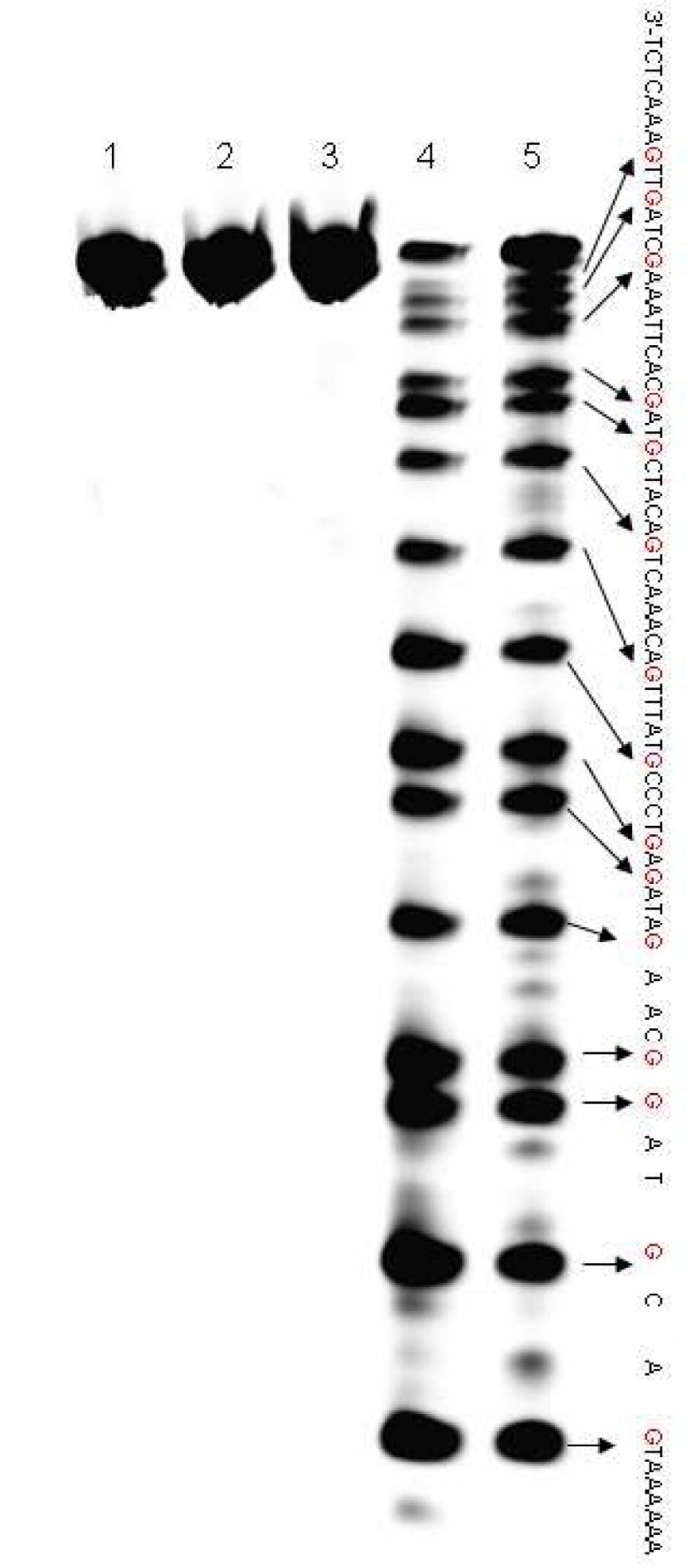
Polyacrylamide gel electrophoresis analysis of 76mer-DNA. The ODN (20 pmol) was oxidized by 20 mM K_2_WO_4_ and 200 mM H_2_O_2_ for 5 min, and further treated with hot piperidine at 90°C for 30 min. Lane 1: ODN alone; lane 2: ODN with 20 mM K_2_WO_4_; lane 3: ODN with 200 mM H_2_O_2_; lane 4: ODN with 20 mM K_2_WO_4_ and 200 mM H_2_O_2_; lane 5: G-ladder (DMS treated).

**Figure 5.**
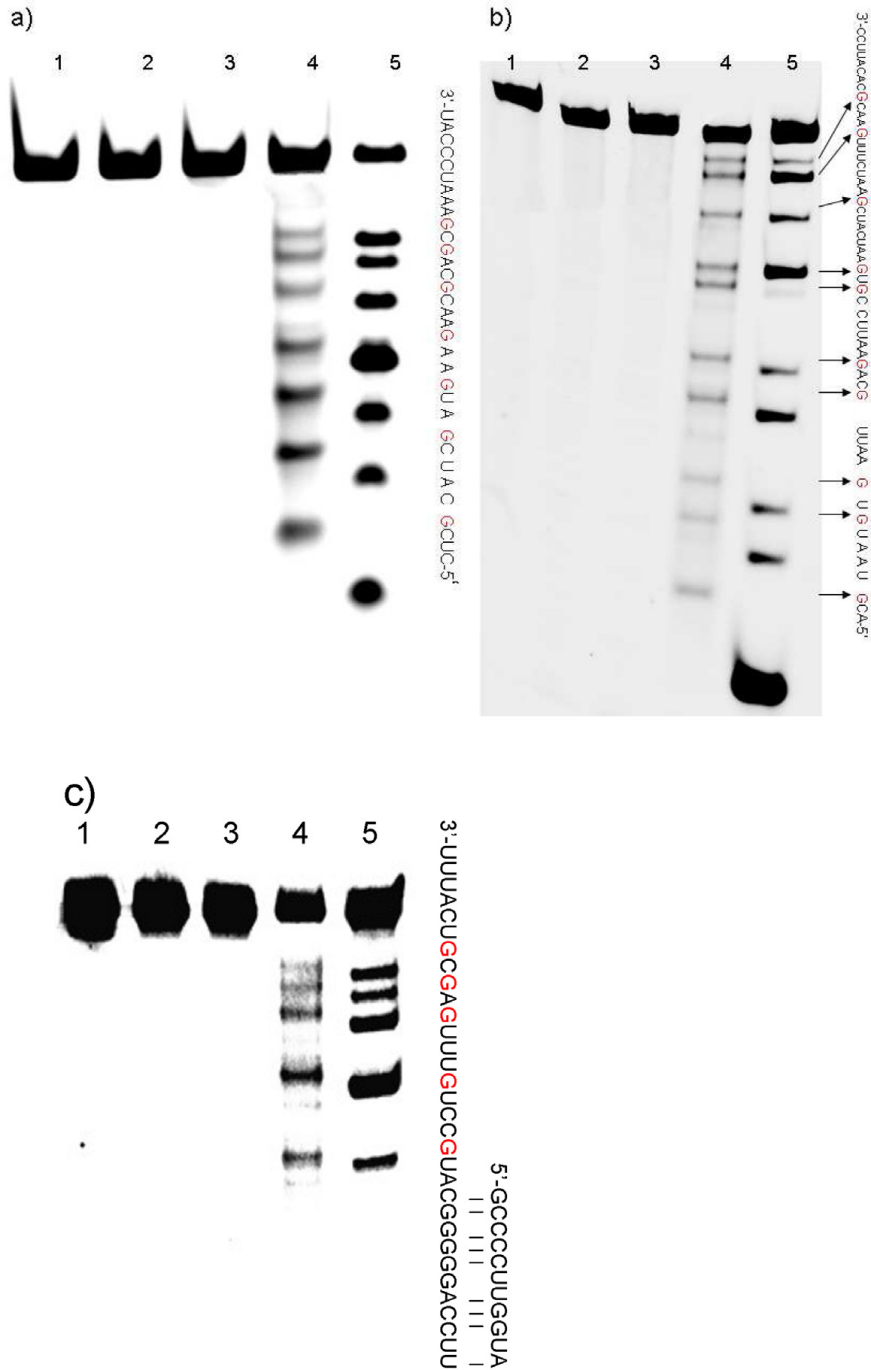
Polyacrylamide gel electrophoresis for oxidized RNA sequences which were incubated with aniline acetate. Treatment of the RNA (20 pmol) with aniline acetate at 60°C for 20 min in the dark after oxidized by 5 mM K_2_WO_4_ and 50 mM H_2_O_2_ for 2 h. Lane 1: RNA alone; lane 2: RNA with 5 mM K_2_WO_4_; lane 3: RNA with 50 mM H_2_O_2_; lane 4: RNA with 5 mM K_2_WO_4_ and 50 mM H_2_O_2_; lane 5: RNA was digested by RNase T1. (**a**) Oxidation of the 33mer-RNA sequence with K_2_WO_4_ and H_2_O_2_. (**b**) Oxidation of the 55mer-RNA sequence with K_2_WO_4_ and H_2_O_2_. (**c**) Oxidation of the 43mer-RNA sequence with K_2_WO_4_ and H_2_O_2_.

**Figure 6.**
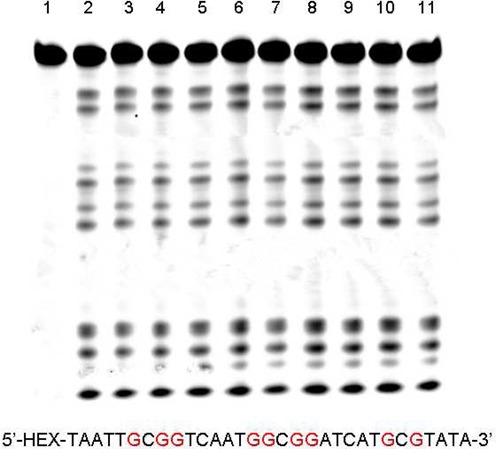
Polyacrylamide gel electrophoresis analysis of 31mer-DNA. Treatment of the ODN (20 pmol) with piperidine at 90°C for 30 min, after oxidized by 20 mM K_2_WO_4_ and 200 mM H_2_O_2_. The reacting time in lanes 1–11 was 0, 1, 2, 3, 4, 5, 6, 7, 8, 9 and 10 min, respectively.

**Figure 7.**
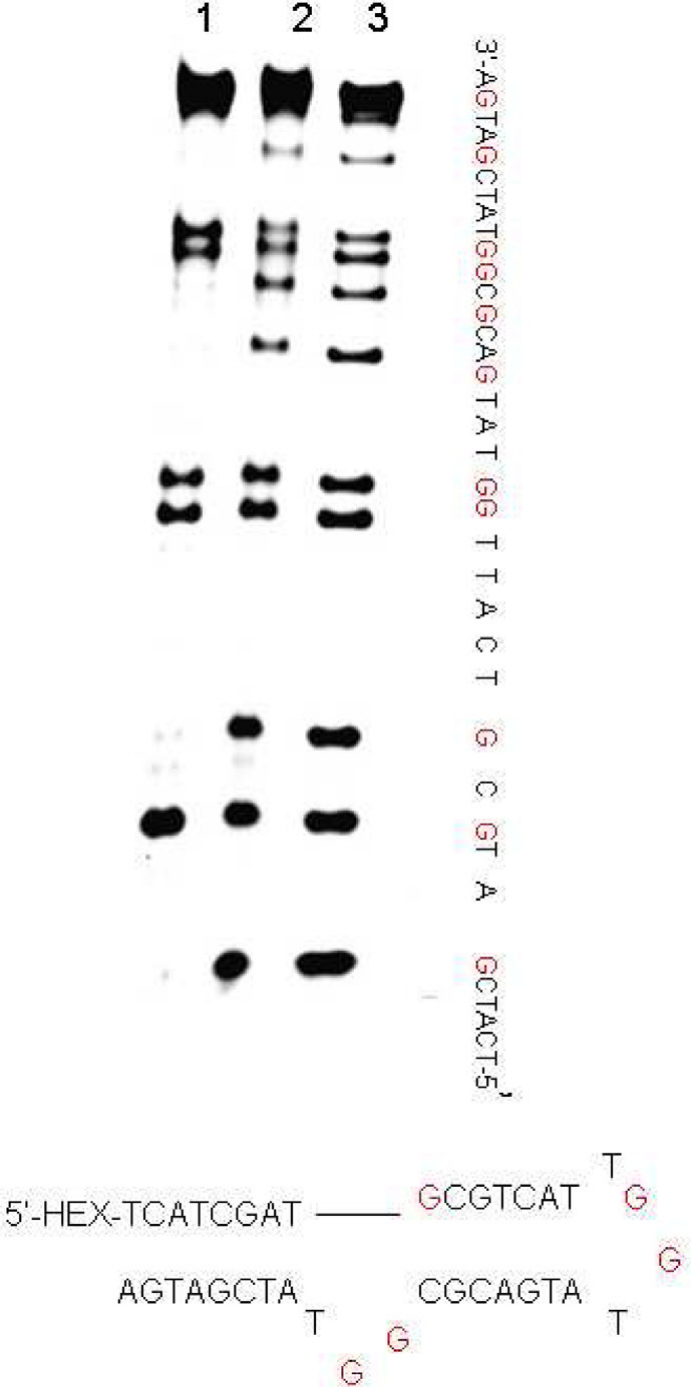
Polyacrylamide gel electrophoresis analysis of 38mer-DNA containing hairpin loop and bulge structures. Lane 1: ODN was oxidized by 20 mM K_2_WO_4_ and 200 mM H_2_O_2_ for 5 min; lane 2: ODN containing 80% formamide was oxidized by 20 mM K_2_WO_4_ and 200 mM H_2_O_2_ for 5 min; lane 3: ODN was treated with DMS.

**Figure 8.**
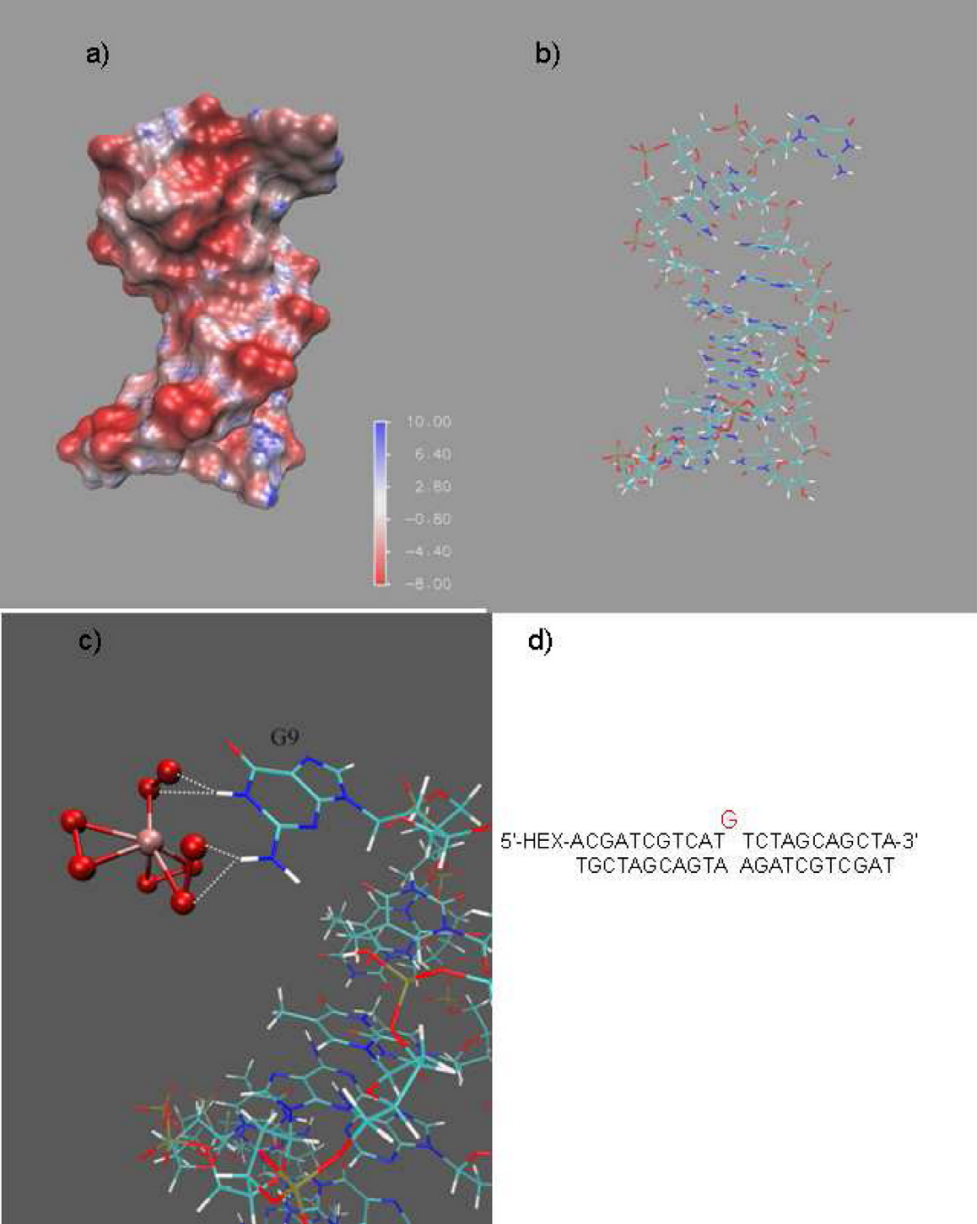
Molecular modeling of bulge structure DNA sequence (DNA 2) and the interaction between K_2_W(O_2_)_4_ and DNA 2. (**a**) Molecular structure of DNA 2. (**b**) Electrostatic surface potential of DNA 2. Value given in units of kT/e. (**c**) The binding location of the bulge domain. All structures are colored by atom type. DNA is painted by lines and W(O_2_)_4_^2−^ is painted in CPK. (**d**) The bulge structure of DNA 2.

First, to investigate whether the scission of the DNA sequence was accomplished by the oxidant alone or the combination of the oxidant and piperidine, polyacrylamide gel electrophoresis (Figure 1) was performed. In Figure 1, the 5’-HEX-labeled ODN 1 was incubated with K_2_WO_4_ and H_2_O_2_ for 2 h in sodium phosphate at 37°C (10 mM, pH 7.0). The subsequent treatment with piperidine revealed important and selective cleavage at the guanosine residue, while no cleavage was observed before the piperidine treatment (Figure 1, lane 8 versus 4). In the absence of K_2_WO_4_ or H_2_O_2_, little reaction was observed (Figure 1, lanes 1, 2, 3, 5, 6, 7). The G-ladder (lane 9) was prepared according to the classical Maxam–Gilbert chemical method for DNA sequencing (20). This experiment indicated that the K_2_W(O_2_)_4_ prepared by the combination of K_2_WO_4_ and H_2_O_2_ can specifically oxidize guanosine, leading to DNA scission under the subsequent hot piperidine treatment. This strategy provided a convenient and efficient method to prepare the G-ladder.

### Analysis of reaction kinetics

In addition, the reaction kinetics, including the reaction time and the K_2_WO_4_ and H_2_O_2_ concentrations, were studied. As shown in Figure 2 and Supplementary Figure S2, the optimum result of this method, with the most obvious cleavage band, was obtained using 5 mM K_2_WO_4_ and 50 mM H_2_O_2_ for 2 h.

### Oxidation of several DNA structures by K_2_WO_4_ and H_2_O_2_

Next, to evaluate the structural information on the non-canonical DNA motifs, our method was applied to seven different representative labeled DNA structures (Figure 3a–g). The DNA sequences were first oxidized with K_2_WO_4_ and H_2_O_2_ and then treated with hot piperidine (Figure 3a–f, lane 4). As expected, a clear band corresponding to guanosine residues was observed in the presence of K_2_WO_4_ and H_2_O_2_ (Figure 3a–f, lane 4). The guanosines in the loop or bulge regions were more sensitive to the specific oxidation (Figure 3a–c), while the guanosines in the duplex region were insensitive to the oxidant (Figure 3g). The guanines in the loop and bulge regions were more exposed to the oxidant when compared with the guanines in Watson–Crick complementary base pairs, favoring the cleavage of the guanosine residues in the loop or bulge (labeled red in the sequences). Therefore, this strategy could be exploited to investigate the complex conformational information of various DNA sequences, with no exceptions for hairpin loops and bulge structures.

Because a high frequency of mismatches occur during DNA replication, which could lead to *in vivo* malfunctions, the mismatch structure has attracted increasing attention in the biochemical field. Here, we studied DNA sequences that react with K_2_WO_4_ and H_2_O_2_, including a mismatch DNA sequence (Figure 3d). In the presence of both K_2_WO_4_ and H_2_O_2_ (Figure 3d, lane 4), an apparent cleavage band was observed in the gel. This cleavage site was located at the mismatched guanosine residue, according to the G-ladder (Figure 3d, lane 5). Therefore, our strategy could be used to probe for mismatched guanosines in a specific DNA sequence. In addition, the terminal guanosine was active in a duplex structure in which every base was paired according to a normal Watson–Crick scheme (Figure 3e). The cleavage band for the terminal guanosine residue (Figure 3e, lane 4) indicated that a particularly accessible guanosine, such as one at the end of a helix, was able to interact with the oxidant. Furthermore, the DNA G-quadruplex structure was also tested (Figure 3f). No cleavage band was observed in the gel, as all the guanines were involved in the formation of the G-quadruplex DNA. Therefore, very little oxidant could oxidize the guanosines in G-quadruplex DNA.

To evaluate whether our method could be applied to longer DNA fragments, a 76mer HEX-labeled DNA was prepared. As expected, the longer DNA sequence was oxidized and subsequently cleaved only in the presence of K_2_WO_4_ and H_2_O_2_ (Figure 4, lane 4). Referring to Figure 4, lane 5 (DMS treated), we could further confirm that only guanosines in the longer DNA sequence were cleaved.

### RNA sequences are oxidized by K_2_WO_4_ and H_2_O_2_

Aside from DNA, we also applied our method to RNA sequences. The guanosines in the RNA sequences were oxidized by K_2_WO_4_ and H_2_O_2_ and then treated with aniline acetate (pH = 4.5). Not surprisingly, these two steps gave rise to cleavage bands that appeared to correspond to guanosine residues, according to the G-ladder prepared with RNase T1 (Figure 5, lane 4 and lane 5). The RNase T1 digestion generated a faster migrating species, including a 3’-phosphate, while the aniline acetate treatment led to the formation of a 3’-protonated Schiff base that retarded migration (29). When we compared oxidized RNA sequences (Figure 5, lane 4) with RNA sequences that were incubated with only RNase T1 (Figure 5, lane 5), the gel experiments confirmed that the guanosines in the RNA sequences were specifically oxidized by K_2_WO_4_ and H_2_O_2_.

To test whether our strategy was suitable for other types of RNA sequences, three RNA sequences with different lengths (33mer, 55mer, 43mer) and structures (ss, ds) were oxidized with K_2_WO_4_ and H_2_O_2_ and incubated with aniline acetate. As expected, the guanosines located in the non-duplex regions were cleaved (Figure 5). The oxidant could not approach any of the guanosines in the duplex regions (Figure 5c). Therefore, our strategy can be used to obtain structural information on RNA containing various secondary structures.

## DISCUSSION

There are numerous reagents to detect guanosine in DNA/RNA, including DMS, RNase T1 and the photooxidation agent—methylene blue. In the classical Maxam–Gilbert chemical method for DNA sequencing, the guanosines can be alkylated specifically and further cleaved by hot piperidine in 5 min. Figure 2 and Supplementary Figure S2 show the results of investigating the reaction kinetics of DNA oxidized by K_2_WO_4_ and H_2_O_2_. We obtained the clearest band using 5 mM K_2_WO_4_ and 50 mM H_2_O_2_ for 2 h. To compete with a classical Maxam–Gilbert reaction using DMS, we tried changing the concentration of oxidants. In the presence of 20 mM K_2_WO_4_ and 200 mM H_2_O_2_, it was possible to obtain higher kinetics for the oxidizing reactions. In Figure 6, we could still observe some clear bands at the guanine residues within as short a time as 0–10 min, which made our method useful for many potential applications. In addition, the guanosines in non-duplex regions were specifically cleaved by K_2_WO_4_ and H_2_O_2_ and piperidine treatment, which was different with DMS and piperidine treatment. In the highest concentration of formamide, our method had the same effect as DMS (Figure 7, Supplementary Figures S3 and S4, lane 2). Thus, our method can not only be applied to double strand DNA sequences, but can slso target the guanine sites in non-duplex regions of nucleic acids. For RNase T1, cleavage occurs between the 3’-phosphate group of a guanine ribonucleotide and the 5’-hydroxyl of the adjacent nucleotide. RNase T1 could specifically cleave RNA at all guanine residues but could not recognize 3’-terminal guanine, which could be made up by the oxidation of K_2_WO_4_ and H_2_O_2._ In Supplementary Figure S6, comparing methylene blue with K_2_WO_4_ and H_2_O_2_, the HEX-labeled DNA was cleaved less by methylene blue (15 min) than by K_2_WO_4_ and H_2_O_2_ (5 min). We could not prolong the photooxidation time because the fluorescent units of DNA were sensitive to the light. Previously, Theodore Friedmann had reported that all guanine residues become sites of strand scission after irradiation and piperidine treatment (24). This photooxidation can occur only in the presence of light and oxygen, which is more complicated than K_2_WO_4_ and H_2_O_2_ and cannot specifically target the guanosines in non-duplex regions. Furthermore, the photooxidation of methylene blue requires alkaline pH (25), while our method can be adapted to different pH values ranging from 3.0 to 9.0 (Supplementary Figure S5). Thus, we have developed a useful method that is less toxic and more widely applicable than RNase T1 and the photooxidation agent methylene blue.

The specific cleavage of guanosine units in non-duplex regions was further investigated through molecular modeling experiments. A bulge DNA 2 structure containing ‘-dG-’ was built with Sybyl 8.1 software (Sybyl software, Version 8.1; St. Louis, Tripos Associates Inc., MO, USA, 2008) (Figure 8a). The electrostatic surface potential of the DNA 2 was calculated using APBS (30) and VMD (31) (Figure 8b). The guanosine units in the bulge region had a higher electrostatic potential energy than the guanosines in the well-matched complementary region. Therefore, the negative W(O_2_)_4_^2−^ anion could easily approach the extruded guanosine units via the electrostatic driving force. A molecular docking experiment of the W(O_2_)_4_^2−^ anion and the DNA using AutoDock 4.2 (32) also supported this hypothesis (Figure 8c). The W(O_2_)_4_^2−^ anion was able to bind to the base portion of the guanosine residue, and the distances between the oxygen atoms of the W(O_2_)_4_^2−^ and the amino and imino groups were approximately 2.09 and 1.90 Å, respectively, thus allowing the transfer of the active oxygen. The binding energy of W(O_2_)_4_^2−^ and DNA is -1.17 kcal/mol, indicating a stable binding conformation. The electrostatic surface potential calculation and docking (Supplementary Figure S7) of DNA 1 showed a similar result, indicating that W(O_2_)_4_^2-^ could also interact with guanosine units in the loop structure.

**Scheme 2.**
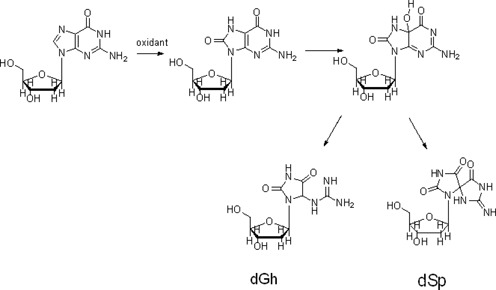
The guanosine could be oxidized to the labile products.

8-Oxo-7, 8-dihydro-2’-guanine (8-oxoG) was the likely intermediate of the guanosine oxidation. Because of its low redox potential, 8-oxoG could be further oxidized to the labile products—dGh and dSp (Scheme 2) (33), which are sensitive to hot piperidine. The MS data for dGh and dSp (Supplementary Figure S1) were consistent with previous reports (33).

In conclusion, we have established a novel approach for the recognition of guanine units in non-duplex nucleic acid regions by potassium tungstate and hydrogen peroxide. We have reported that potassium tungstate and hydrogen peroxide can specifically cleave guanosine units in single strand DNA. This ability provides an effective agent for sequencing guanosines. In addition, our strategy can specifically cleave guanosine units in loops, bulge, terminal and mismatch structures. These results indicated that our strategy is applicable to study the conformational information of various DNAs containing different secondary structures. Furthermore, the guanosines in non-duplex RNA regions were also cleaved specifically. These results illustrated that our strategy could be used to study the structures of various RNA sequences.

## SUPPLEMENTARY DATA

Supplementary Data are available at NAR Online.

SUPPLEMENTARY DATA
