# Supplementary material for: Specific recognition of guanines in non-duplex regions of nucleic acids with potassium tungstate and hydrogen peroxide
Source: Nucleic Acids Res. 2014 Oct 29;43(1):e3. doi: 10.1093/nar/gku1025 (PMC4288145; doi:10.1093/nar/gku1025)
Supplement: SUPPLEMENTARY DATA [file supp_43_1_e3__index.html]

Specific recognition of guanines in non-duplex regions of nucleic acids with potassium tungstate and hydrogen peroxide — SUPPLEMENTARY DATA 

# Specific recognition of guanines in non-duplex regions of nucleic acids with potassium tungstate and hydrogen peroxide

## SUPPLEMENTARY DATA

**Files in this Data Supplement:**

- SUPPLEMENTARY DATA
