# Supplementary material for: Specific recognition of guanines in non-duplex regions of nucleic acids with potassium tungstate and hydrogen peroxide
Source: Nucleic Acids Res. 2014 Oct 29;43(1):e3. doi: 10.1093/nar/gku1025 (PMC4288145; doi:10.1093/nar/gku1025)
Supplement: SUPPLEMENTARY DATA [file supp_gku1025_nar-02317-met-f-2014-File016.doc]

**Supplementary Information**

**Specifically Recognition of Guanine Site in Non-duplex Regions of Nucleic Acids by** **Potassium Tungstate and Hydrogen Peroxide**

Wuxiang Mao, Xiaowei Xu, Huan He, Rong Huang, Xi Chen, Heng Xiao, Zhenduo Yu, Yi Liu and Xiang Zhou*


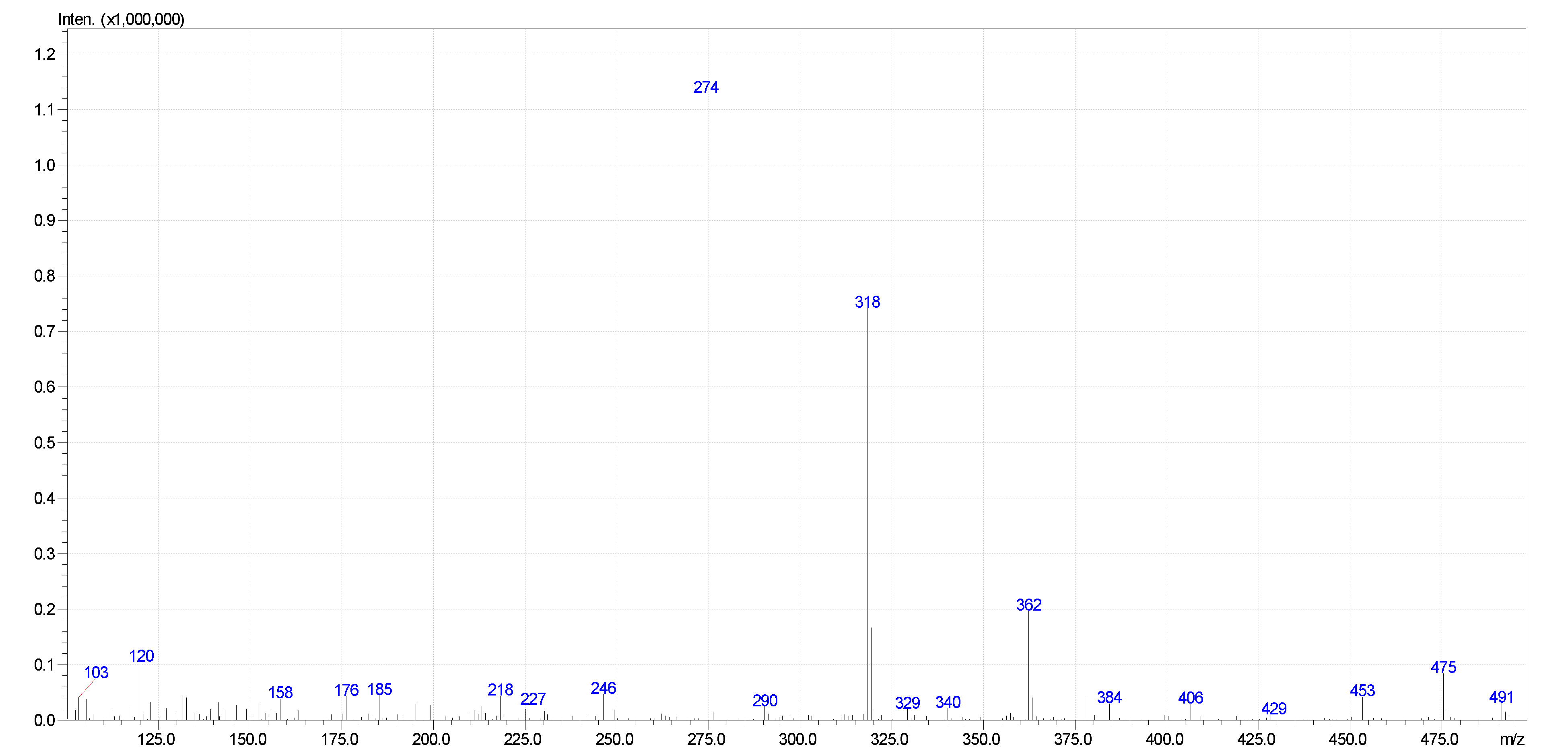


**Figure S1** ESI^+^-MS analysis for nucleoside products observed from dG oxidation with K_2_WO_4_ and H_2_O_2_ on the C18 RP-HPLC column. m/e “274”=dGh+H^+^, m/e “318”=dSp+H_2_O+H^+^.


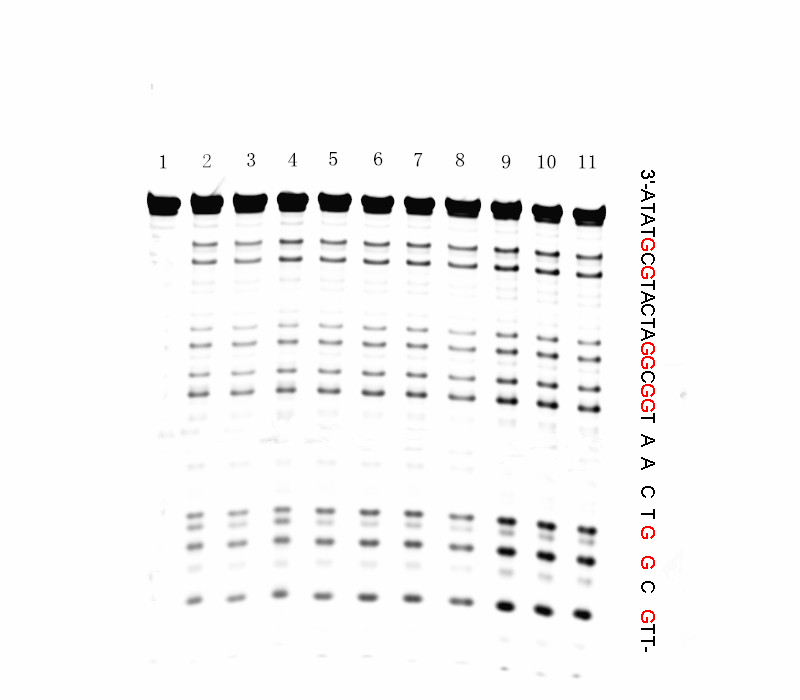


**Figure S2** Polyacrylamide gel electrophoresis analysis of ODN 1. Treat of the ODN 1 (20pmol) with piperidine at 90 °C for 30min, after oxidized by 5 mM K_2_WO_4_ and 50 mM H_2_O_2_, The reacting time in lanes 1-11 was 0 h, 0.25 h, 0.5 h, 0.75 h, 1 h, 1.25 h, 1.5 h, 1.75 h, 2 h, 2.25h and 2.5h, respectively.


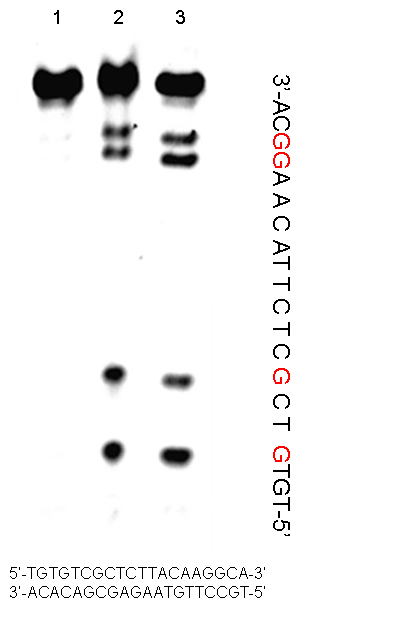


**Figure S3** Polyacrylamide gel electrophoresis analysis of ds-DNA. Lane 1: ds-DNA was oxidized by 20 mM K_2_WO_4_ and 200 mM H_2_O_2_ for 5min; Lane 2: ds-DNA containg 80% formamide was oxidized by 20 mM K_2_WO_4_ and 200 mM H_2_O_2_ for 5min; Lane 3: ds-DNA was treated with DMS.


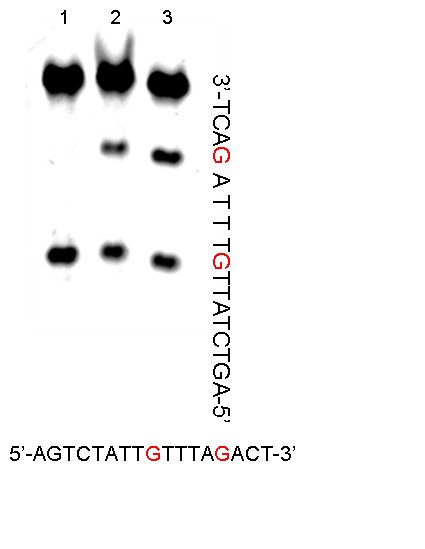


**Figure S4** Polyacrylamide gel electrophoresis analysis of 17 mer-hairpin loop(DNA 1). Lane 1: DNA 1 was oxidized by 20 mM K_2_WO_4_ and 200 mM H_2_O_2_ for 5min; Lane 2: DNA 1 containg 80% formamide was oxidized by 20 mM K_2_WO_4_ and 200 mM H_2_O_2_ for 5min; Lane 3: DNA 1 was treated with DMS.


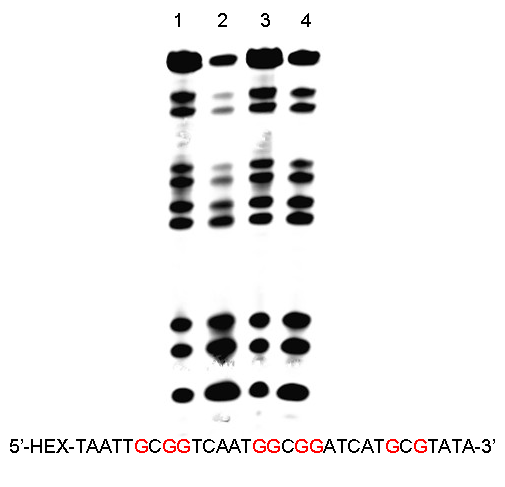


**Figure S5** Polyacrylamide gel electrophoresis analysis of 31mer-ODN1, which was oxidized in Na_2_HPO_4_-NaH_2_PO_4_ buffer at different pH values. Lane 1: ODN 1 was oxidized by 20 mM K_2_WO_4_ and 200 mM H_2_O_2_ for 5min in Na_2_HPO_4_-NaH_2_PO_4_ buffer( pH 3.0) ; Lane 2: ODN 1 was oxidized by 20 mM K_2_WO_4_ and 200 mM H_2_O_2_ for 5min in Na_2_HPO_4_-NaH_2_PO_4_ buffer( pH 5.0); Lane 3: ODN 1 was oxidized by 20 mM K_2_WO_4_ and 200 mM H_2_O_2_ for 5min in Na_2_HPO_4_-NaH_2_PO_4_ buffer( pH 7.0); Lane 4: ODN 1 was oxidized by 20 mM K_2_WO_4_ and 200 mM H_2_O_2_ for 5min in Na_2_HPO_4_-NaH_2_PO_4_ buffer( pH 9.0).


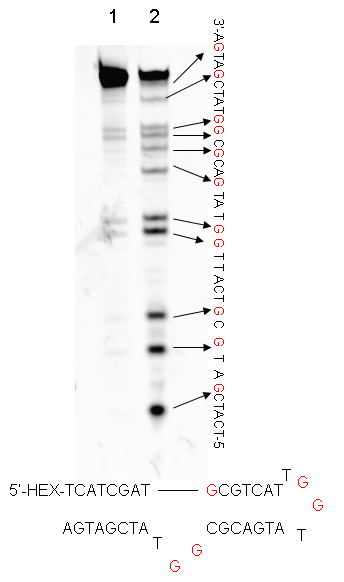


**Figure S6** Polyacrylamide gel electrophoresis analysis of 38mer-DNA containig hairpin loop and bulge structure. Lane 1: DNA was treated with methylene blue in the presence of light and oxygen for 15 minutes; Lane 2: DNA( degenerated) was oxidized by 20 mM K_2_WO_4_ and 200 mM H_2_O_2_ in 80% formamide for 5 minutes.


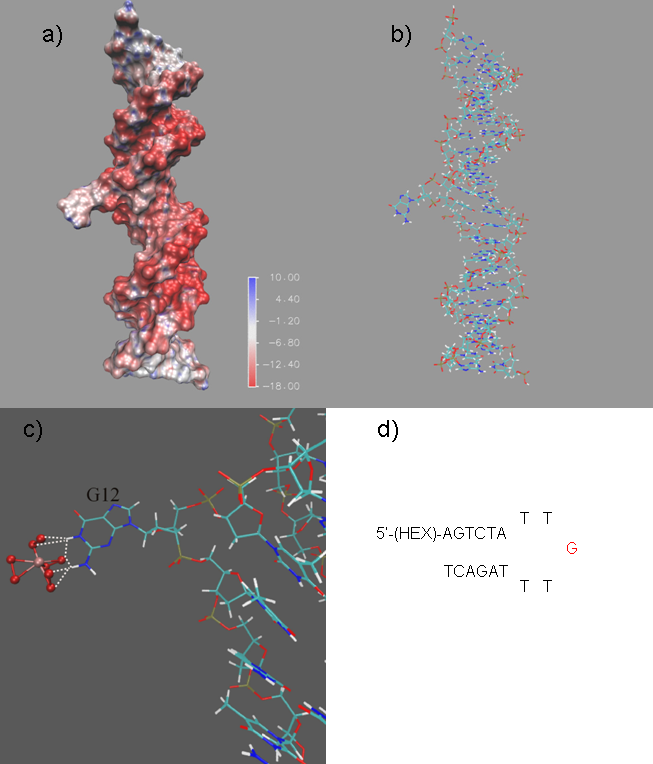


**Figure S7** Molecular modeling of hairpin loop structure DNA sequence (DNA 1) and the interaction between K_2_W(O_2_)_4_ and DNA 1. a) Electrostatic surface potential of DNA 1. Value given in units of kT/e. b) Molecular structure of DNA1. c) The binding location of on the loop domain. All structure are colored by atom type. DNA is painted by lines and W(O_2_)_4_^2-^ is painted in CPK. d) The loop structure of DNA 1.

**Nucleic acid sequences:**

ODN1: 5’-HEX-TAATTGCGGTCAATGGCGGATCATGCGTATA-3’

hairpin loop(DNA 1): 5’-HEX-AGTCTATTGTTTAGACT-3’

bulge(DNA 2): 5’-HEX-ACGATCGTCATGTCTAGCAGCTA-3’

3’- TGCTAGCAGTA -AGATCGTCGAT-5’

loop and bulge: 5’-HEX-TCATCGATGCGTCATTGGTACTGACGCGGTATCGATGA-3’

mismatch: 5’-HEX-ACGATCGTCATGTCTAGCATCTA-3’

3’- TGCTACGAGTAGAGATCGTAGAT-5’

terminal: 5’-HEX-CATGCGTTCCCGTG-3’

3’-GTACGCAAGGGCAC-5’

G-qudruplex: 5’-HEX-TTAGGGTTAGGGTTAGGGTTAGGG-3’

ds-DNA: 5’-TGTGTCGCTCTTACAAGGCA-3’

3’-ACACAGCGAGAATGTTCCGT-5’

76mer-DNA:5’-HEX-AAAAAATGACGTAGGCAAGATAGAGTCCCGTATTTGACAAACTGACATCGTAGCACTTAAAGCTAGTTGAAACTCT-3’

33mer-RNA: 5’-HEX-CUCGCAUCGAUGAAGAACGCAGCGAAAUCCCAU-3’

55mer-RNA:5’-HEX-ACGUAAUGUGAAUUGCAGAAUUCCGUGAAUCAUCGAAUCUUUGAACGCACAUUCC-3’

43 mer-RNA: 5’-HEX-GCCCCUUGGUAUUCCAGGGGGCAUGCCUGUUUGAGCGUCAUUU-3’
